# A phase II trial of an alternative schedule of palbociclib and embedded serum TK1 analysis

**DOI:** 10.1038/s41523-022-00399-w

**Published:** 2022-03-21

**Authors:** Jairam Krishnamurthy, Jingqin Luo, Rama Suresh, Foluso Ademuyiwa, Caron Rigden, Timothy Rearden, Katherine Clifton, Katherine Weilbaecher, Ashley Frith, Anna Roshal, Pavan K. Tandra, Mathew Cherian, Tracy Summa, Brittney Haas, Shana Thomas, Leonel Hernandez-Aya, Mattias Bergqvist, Lindsey Peterson, Cynthia X. Ma

**Affiliations:** 1 Division of Oncology/Hematology, University of Nebraska Medical Center, Omaha, NE USA; 2 Division of Public Health Science, Department of Surgery, Siteman Cancer Center, Biostatistics Shared Resource, Washington University School of Medicine, St. Louis, MO USA; 3 Divison of Oncology, Department of Medicine, Washington University School of Medicine, St. Louis, MO USA; 4 Biovica International AB, Uppsala, Sweden; 5 Present Address: Division of Oncology, Department of Medicine, The Ohio State University, Columbus, OH USA

**Keywords:** Breast cancer, Metastasis

## Abstract

Palbociclib 3-weeks-on/1-week-off, combined with hormonal therapy, is approved for hormone receptor positive (HR+)/HER2-negative (HER2−) advanced/metastatic breast cancer (MBC). Neutropenia is the most frequent adverse event (AE). We aim to determine whether an alternative 5-days-on/2-days-off weekly schedule reduces grade 3 and above neutropenia (G3 + ANC) incidence. In this single-arm phase II trial, patients with HR+/HER2− MBC received palbociclib 125 mg, 5-days-on/2-days-off, plus letrozole or fulvestrant per physician, on a 28-day cycle (C), as their first- or second-line treatment. The primary endpoint was G3 + ANC in the first 29 days (C1). Secondary endpoints included AEs, efficacy, and serum thymidine kinase 1 (sTK1) activity. At data-cutoff, fifty-four patients received a median of 13 cycles (range 2.6–43.5). The rate of G3 + ANC was 21.3% (95% CI: 11.2–36.1%) without G4 in C1, and 40.7% (95% CI: 27.9–54.9%), including 38.9% G3 and 1.8% G4, in all cycles. The clinical benefit rate was 80.4% (95% CI: 66.5–89.7%). The median progression-free survival (mPFS) (95% CI) was 19.75 (12.11–34.89), 33.5 (17.25–not reached [NR]), and 11.96 (10.43–NR) months, in the overall, endocrine sensitive or resistant population, respectively. High sTK1 at baseline, C1 day 15 (C1D15), and C2D1 were independently prognostic for shorter PFS (*p* = 9.91 × 10^−4^, 0.001, 0.007, respectively). sTK1 decreased on C1D15 (*p* = 4.03 × 10^−7^), indicating target inhibition. Rise in sTK1 predicted progression, with the median lead time of 59.5 (inter-quartile range: −206.25–0) days. Palbociclib, 5-days-on/2-days-off weekly, met its primary endpoint with reduced G3 + ANC, without compromising efficacy. sTK1 is prognostic and shows promise in monitoring the palbociclib response. ClinicalTrials.gov#: NCT3007979.

## Introduction

Hormone receptor positive (HR+) and human epidermal growth factor receptor 2 negative (HER2−) breast cancer accounts for ~70% of breast cancer diagnoses and is a leading cause of cancer death in women^1,2^. The discovery of cyclin D/cyclin-dependent kinase 4/6 (CDK4/6) as a key downstream target of estrogen receptor (ER) and endocrine resistance mechanisms has led to the development of CDK4/6 inhibitors for the treatment of HR+/HER2− breast cancer^3,4^. CDK4/6 inhibitors, including palbociclib, ribociclib, and abemaciclib, have gained FDA approval based on the significant improvement in progression-free survival (PFS) when added to endocrine therapy in patients with advanced disease as front-line therapy^5–11^ or following disease progression on prior endocrine therapy^12–14^. Overall survival (OS) benefit has also been observed in several studies^15–17^. These agents have now become the standard of care in combination with an endocrine therapy partner for HR+/HER2− metastatic breast cancer (MBC). However, treatment-related neutropenia is a common adverse event (AE), leading to dose interruption, reduction, and at times discontinuation. Palbociclib (Ibrance, Pfizer) is the first CDK4/6 inhibitor approved in combination with an aromatase inhibitor as first-line therapy and with fulvestrant following prior hormonal therapy based on results from PALOMA trials^5,9,10,13^. Neutropenia was the most frequent high grade (G) AE related to palbociclib in PALOMA-2 (79.5% all grades, 56.1% G3, 10.4% G4) and PALOMA-3 (78.8% all grades, 53.3% G3, 8.7% G4)^9,13^. Febrile neutropenia occurred in 1.8%, while dose reduction occurred in over a third of patients who received palbociclib across PALOMA-2 and PALOMA-3^9,13,18^. In addition, with the half-life of palbociclib being ~27 h, recovery of Rb phosphorylation and cell proliferation during the off-treatment week is a concern. In a longitudinal biomarker study of palbociclib plus letrozole, recovery of tissue Rb phosphorylation and Ki67 levels to baseline was observed on day 3 and day 4–5 during the off week, respectively^19^. We, therefore, proposed a 5-days-on/2-days-off weekly schedule to allow bone marrow recovery during the 2 off days to avoid the week-long break. We hypothesize that this alternative schedule is more tolerable with less frequent high-grade (G3+) neutropenia and dose interruptions/reductions compared to the historical data with the 3-weeks-on/1-week-off schedule. Based on our previous study supporting serum thymidine kinase 1 (sTK1), an E2F-dependent enzyme critical for DNA synthesis, as a pharmacodynamic indicator of CDK4/6 inhibition^20^, we assessed sTK1 dynamics for target inhibition, and examined its potential prognostic value and utility in disease monitoring in this study.

## Results

Between 12 July 2017 and 14 Feb 2020, 54 patients with HR+/HER2− MBC were enrolled to receive palbociclib plus letrozole (*n* = 41) or fulvestrant (*n* = 13) per physician’s choice (Table 1). Premenopausal women (*n* = 8) also received goserelin. Twenty-two (41%) patients had primary or secondary endocrine resistance by ESMO guideline (footnote of Table 1)^21^. As of the data cutoff date of 15 Dec 2020, at a median follow-up of 27.04 (95% CI: 23.89–41.82) months, treatment is ongoing for 19 (35%) patients. A majority (17/54, 31%) of patients went off study due to progression (Supplementary Fig. 1).

**Table 1:** Clinical Characteristics (*n* = 54).

Characteristics	*N* (%)
Age, years	
Median (range)	61 (34–87)
Race	
White	45 (83%)
African American	9 (17%)
ECOG PS	
0	29 (54%)
1	21 (39%)
2	4 (7%)
Menopausal status	
Premenopausal	8 (15%)
Postmenopausal	46 (85%)
Endocrine drug partner	
Letrozole	41 (76%)
Fulvestrant	13 (24%)
Visceral metastases	
Yes	28 (52%)
Prior therapy	
Neo/adjuvant chemotherapy	22 (41%)
Neo/adjuvant endocrine therapy	29 (54%)
Metastatic chemotherapy	0 (0%)
Metastatic endocrine therapy	4 (7%)
Setting of trial therapy, endocrine sensitivity^a^	
1st line, de novo stage IV, endocrine sensitive	20 (37%)
1st line, recurrent stage IV, endocrine sensitive	12 (22%)
1st line, primary endocrine resistance	1 (2%)
1st line, secondary endocrine resistance	17 (31%)
2nd line, primary endocrine resistance	1 (2%)
2nd line, secondary endocrine resistance	3 (6%)

^a^Per ESMO guideline (Cardoso et al.^21^), defined as the following:

Endocrine sensitive: relapse at least 12 months following completing of adjuvant endocrine therapy or with de novo MBC.

Primary endocrine resistance: relapse while on the first 2 years of adjuvant endocrine therapy, or PD within the first 6 months of 1st line endocrine therapy for advanced breast cancer, while on endocrine therapy

Secondary endocrine resistance: relapse while on adjuvant endocrine therapy but after the first 2 years, or relapse within 12 months of completing adjuvant endocrine therapy, or PD ≥ 6 months after initiating endocrine therapy for advanced breast cancer, while on endocrine therapy.

Forty-seven patients were evaluable for the primary endpoint of G3 + ANC during C1D1-29 (Supplementary Figure 1). Ten (21.3%, 95% CI: 11.2–36.1%) had G3 ANC, without G4 AE, in C1D1-29 (Table 2). As a pooled analysis of PALOMA trials published recently indicted a G3 + ANC rate of ~45%^18^, we performed a post hoc power calculation that tested the observed 21.3% rate against the null hypothesis >44.7%. With *N* = 47, the post hoc power is 94.04% based on a 1-sided Binomial exact test at 5% alpha level.

**Table 2:** Adverse events.

AE	G1	G2	G3	G4	Total
C1 D1-29 (*n* = 47)					
Non-hematologic AE					
Fatigue	13 (27.7%)	0	0	0	13 (28%)
Hot flashes	7 (14.9%)	0	0	0	7 (15%)
Nausea	6 (12.8%)	1 (2.1%)	0	0	7 (15%)
Alopecia	5 (10.6%)	0	0	0	5 (11%)
Dizziness	1 (2.1%)	0	1 (2.1%)	0	2 (4%)
Laboratory investigation					
Leukopenia	13 (27.6%)	22 (46.8%)	7 (14.9%)	0	42 (89%)
Neutropenia	6 (12.8%)	18 (38.3%)	10 (21.3%)	0	34 (72%)
Anemia	18 (38.3%)	5 (10.6%)	0	0	23 (49%)
Lymphopenia	6 (12.8%)	8 (17%)	2 (4.3%)	0	16 (34%)
Thrombocytopenia	5 (10.6%)	1 (2.1%)	0	0	6 (13%)
All cycles (*n* = 54)					
Non-hematologic AE					
Fatigue	20 (37.0%)	4 (7.4%)	0	0	24 (44.4%)
Nausea	19 (35.2%)	3 (5.6%)	0	0	22 (40.7%)
Alopecia	19 (35.2%)	0	0	0	19 (35.2%)
Dizziness	4 (7.4%)	0	1 (1.9%)	0	5 (9%)
Sepsis	0	0	1 (1.9%)	0	1 (1.9%)
Weight loss	1 (1.9%)	0	1 (1.9%)	0	2 (3.8%)
Laboratory investigation					
Leukopenia	7 (13.0%)	21 (38.9%)	23 (42.6%)	0	51 (94.5%)
Neutropenia	2 (3.7%)	24 (44.4%)	21 (38.9%)	1 (1.9%)	48 (88.9%)
Anemia	27 (50.0%)	11 (20.4%)	4 (7.4%)	0	42 (77.8%)
Lymphopenia	6 (11.1%)	18 (33.3%)	11 (20.4%)	0	35 (64.8%)
Thrombocytopenia	17 (31.5%)	0	1 (1.9%)^a^	0	18 (33.3%)
ALT elevated	3 (5.6%)	0	2 (3.7%)	0	5 (9%)
AST elevated	4 (7.4%)	0	1 (1.9%)	0	5 (9%)

All grade 3 AEs, AEs > 10% incidence in the first 29 days, and AEs > 15% incidence all cycles are included.

^a^Patient died from subdural hematoma (G5).

Twenty-two of 54 (40.7%; 95% CI: 27.9–54.9%) had G3 (*n* = 21) or G4 (*n* = 1) ANC in all cycles. Non-hematologic toxicities were uncommon and mostly G1/G2 (Table 2). There was no neutropenic fever. Among the 51 patients who completed at least 1 cycle of therapy, the median dose intensity was 97% (range 54–100%) with a median of 14 (2–44) months of treatments (Table 3). Palbociclib was dose reduced to 100 mg in 12 (24%) patients due to G3 ANC (*n* = 8), thrombocytopenia (*n* = 1), physician decision (*n* = 1), or G3 aspartate aminotransferase/alanine aminotransferase (AST/ALT) (*n* = 2), then further reduced to 75 mg in 8 of these 12 patients due to G3 ANC (*n* = 6), ALT (*n* = 1), thrombocytopenia (*n* = 1). Three (68%) of these 51 patients discontinued due to AE, including 2 due to G3 ANC, and 1 death (G5) due to subdural hematoma, with platelet count of 59 K. There were no other G5 events.

**Table 3:** Exposure to palbociclib.

Exposure to palbociclib (*n* = 51)	
Treatment duration, month	
Median (range)	14 (2–44)
Relative dose intensity^a^	
Median (range)	97% (54%–100%)
Mean (Standard deviation)	90% (14%)
Dose modifications (any cause)	
Dose reductions, *N* (%)	
≥1 dose reduction	12 (24%)
1 dose reduction	4 (8%)
2 dose-level reduction	8 (16%)
Discontinuation due to AE	
*N* (%)	3 (6%)
Dose Interruptions due to AE	
*N* (%)	33 (65%)

^a^Relative dose intensity = [(actual dose)/ (intended dose)]*100%.

**Fig. 1:**
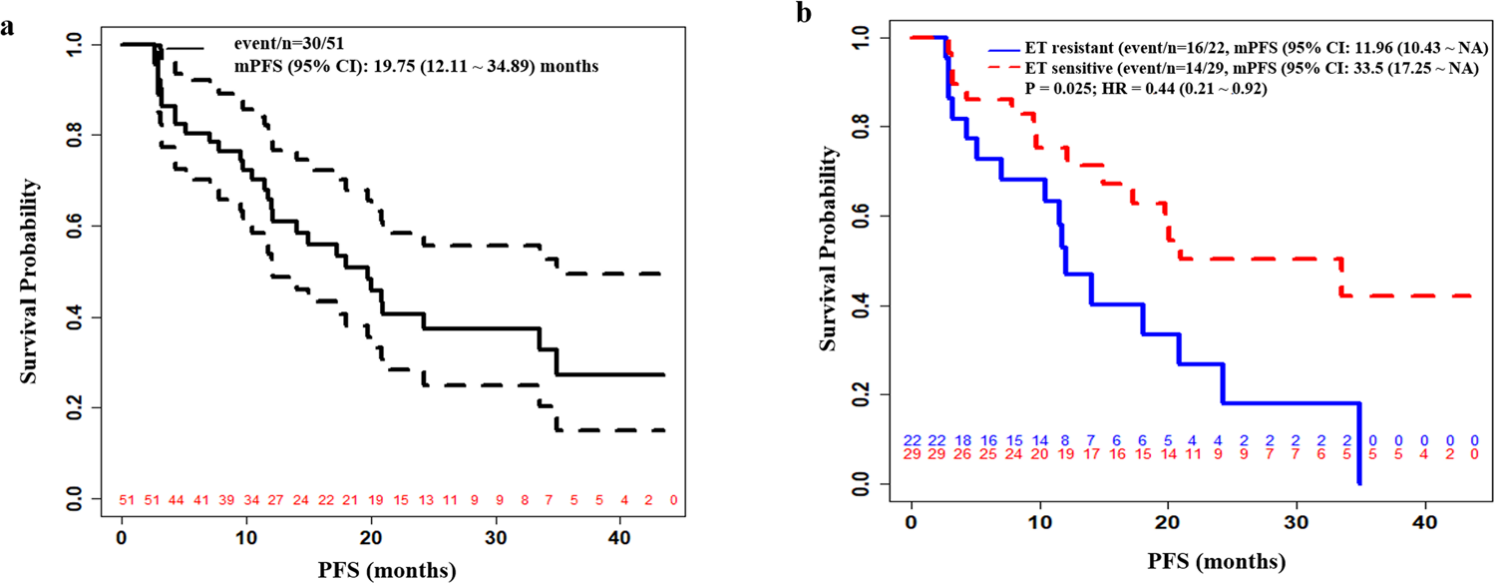
Progression-free survival (PFS) of all treated patients. **a** the overall population, and **b** by endocrine sensitivity.

Clinical benefit at 24 weeks was observed in 41 of 51 evaluable patients, with a CBR of 80.4% (95% CI: 66.5–89.7%), including 2 CR, 14 PR, and 25 SD ≥ 24 weeks (Supplementary Table 1). ORR was 53.3% (95% CI: 34.6–71.2%) in the 30 patients with measurable disease (*n* = 29) or with non-measurable disease but experienced CR (*n* = 1). The mPFS was 19.75 months (95% CI: 12.11–34.89) overall (Fig. 1a), 11.96 (95% CI: 10.43~NR) and 33.5 months (95% CI: 17.25~NR) in the endocrine resistant, or sensitive population, respectively (Fig. 1b).

**Fig. 2:**
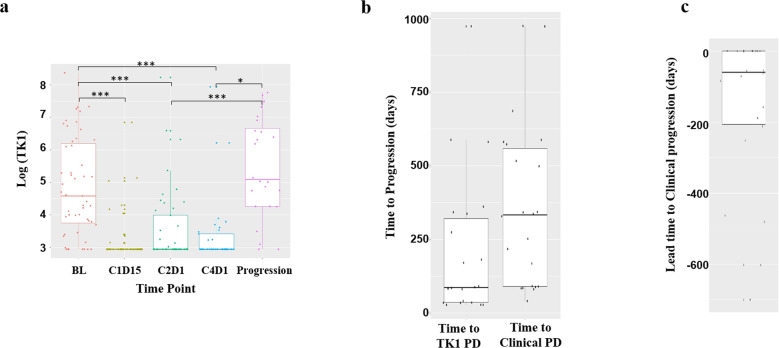
Serum TK1 activity over time. **a** Box plot of log (sTK1 Du/L) along the 5-time points indicated. **b** Time to progression by TK1 increase from the previous time point (sTK1 PD) or by RECIST/investigator decision (Clinical PD). **c** The lead time from TK1 progression to clinical/RECIST progression. BL baseline, C1D15 cycle 1 day 15, C2D1 cycle 2 day 1, Progression PFS events that included RECIST progression (*n* = 17) and investigator decision (*n* = 7). The center horizontal line of the box plot indicates the median, while the box limits indicate the upper and lower quartiles. The box-plot whiskers show a 1.5× interquartile range. Those points outside the whisker line are indicated as outliers. *p < 0.05, ***p < 0.001.

To assess treatment-induced pharmacodynamics effect, we analyzed sTK1 activity on serial blood collected at baseline (*n* = 51), C1D15 (*n* = 46), C2D1 (*n* = 45), C4D1 with 1st tumor imaging (*n* = 33), and at progression for 24 patients who had RECIST progression (*n* = 17) or by investigator decision (*n* = 7). All available samples were tested. 16 of 17 patients with RECIST progression had serial sTK1 assayed every ~3 cycles until progression. The median (interquartile range) sTK1 was 97.87 (42.4–490.3) Du/L at baseline, reduced to <20 (<20~<20) Du/L at C1D15, remained low at <20 (<20~54.1) Du/L at C2D1 and <20 (<20~30.5) Du/L at C4D1 (excluding patients progressed at C4D1), and increased to 162.2 (70.7~793.1) Du/L at disease progression (Fig. 2a, Supplementary Fig. 2, Supplementary Table 2). Comparison of sTK1 levels between selected time points are shown in Supplementary Table 3. sTK1 was undetectable (<20 Du/L) in 13.7% (7/51) at baseline, 78.2% (36/46) at C1D15, 63.6% (28/44) at C2D1, and 8.3% (2/24) at progression. All patients with high sTK1 at C1D15 (≥20 Du/L) had high sTK1 at baseline (>200 Du/L, predefined cut point for high baseline sTK1). Among the 24 patients who progressed with sTK1 available, an sTK1 increase from the prior time point was detected at the time of progression in nine patients, and at an earlier time point in 13 patients (Supplementary Table 4), with the median (inter-quartile range) lead time of 59.5 (−206.25–0) days, Wilcoxon signed rank test *p* = 0.0017 (Fig. 2a, c and Supplementary Table 4).

**Fig. 3:**
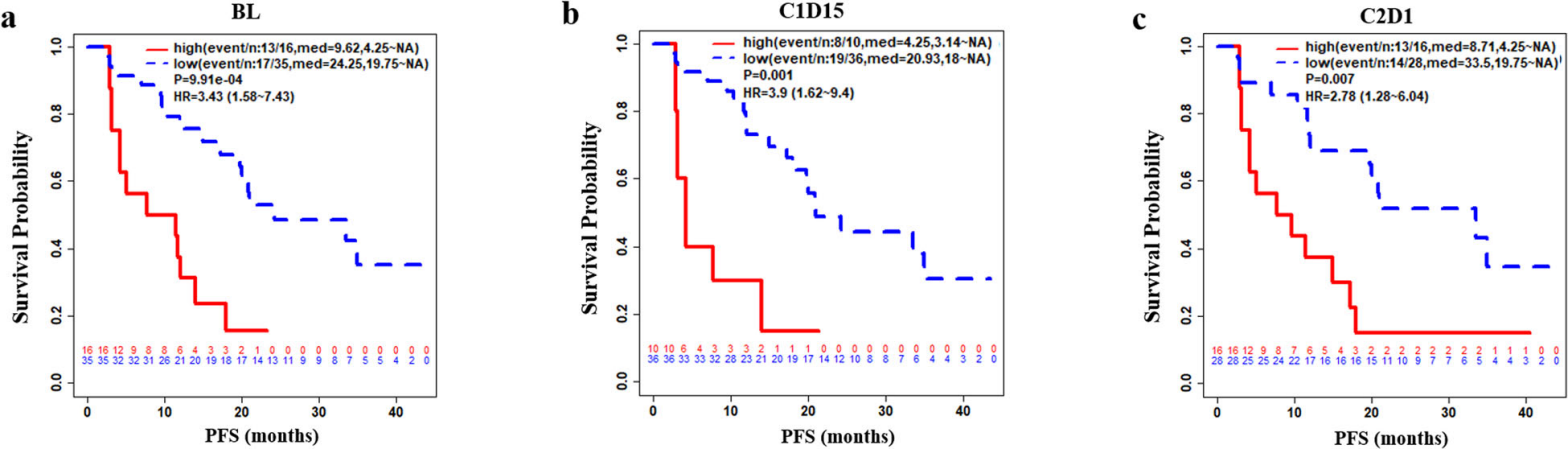
PFS by sTK1 and time points of baseline (BL), C1D15, C2D1, C4D1. sTK1 cutoff for high versus low is 200 at BL (**a**) and 20 at time points C1D15 (**b**) and C2D1 (**c**). Shown in legends are even/n (number of events/total samples), median PFS, 95% CI by group, log rank test P, and HR (hazard ratio: high sTK1 vs. low sTK1) with 95% CI. The number of patients at risk is indicated at the bottom of each plot.

We then assessed the prognostic significance of baseline and early on-treatment sTK1. There was no association between levels of sTK1 either at baseline or C1D15 with the presence of visceral metastasis or endocrine sensitivity defined by ESMO criteria (Supplementary Tables 5, 6). However, sTK1 at both BL and C1D15 time points were significantly correlated with clinical benefit versus not (Supplementary Tables 5, 6). Baseline sTK1 was higher in patients with PD as the best response (Supplementary Fig. 3a; Supplementary Table 5). Progression within 6 months (not achieving clinical benefit) was negatively correlated with high sTK1 at baseline (*p* = 0.000419) and C1D15 (*p* = 8.4 × 10^−5^) (Supplementary Fig. 3b, c). High sTK1 at baseline (>200 Du/L), C1D15 (>20 Du/L) or C2D1 (>20 Du/L) predicted progression within 3 (or 6) months with a specificity of 71.1% (78.0%), 82.5% (89.2%), 65.8% (73.5%), and sensitivity of 50% (70%), 50% (66.7%), 50% (70%), negative predictive value (NPV) of 91.4% (91.4%), 91.6% (91.7%), and 89.3% (89.3%), and positive predictive value (PPV) of 18.8% (43.8%), 30% (60%), and 18.8% (43.8%), respectively (Supplementary Table 7). High baseline sTK1 (>200 Du/L) was negatively correlated with PFS (*p* = 9.91 × 10^−4^), as was incomplete suppression of sTK1 (≥20 Du/L) at an early on-treatment time point at C1D15 (*p* = 0.001) or C2D1 (*p* = 0.007) (Fig. 3). The mPFS (95% CI) for patients with high (≥20 Du/L) sTK1 (*n* = 10) or low sTK1 (*n* = 36) at C1D15 was 4.25 (3.14~NA) and 20.93 (18~NA) months, respectively (HR = 3.9, *p* = 0.001) (Fig. 3). sTK1 at BL, C1D15, and C2D1 remained an independent predictor of PFS in the multivariate analysis that included age, endocrine sensitivity, and sites of metastases (Supplementary Table 8).

## Discussion

CDK 4/6 inhibitors represent a major advance in the treatment of HR+/HER2− MBC. However, a one-week break following 3 weeks of administration is required for both palbociclib and ribociclib due to treatment-induced neutropenia^22,23^. To improve the tolerability and to avoid the 1-week break, which has been shown to led to the recovery of target inhibition in previous studies^19^, we tested palbociclib at 125 mg in a 5-days-on/2-days-off weekly schedule, in combination of letrozole or fulvestrant per physician’s choice as 1st line or 2nd line systemic therapy in this phase II trial in patients with HR+/HER2− MBC. In this trial, we observed G3 + ANC rate of 21.3% (95% CI: 11.2%–36.1%, without G4) in D1-29, and 40.7% (95% CI: 27.9%–54.9%) (1.9% G4) in all cycles with a median dose intensity of 97% (range 54–100%) in a median of 14 months of drug therapy. Dose reduction occurred in 24% of patients, including 6% who discontinued therapy. The trial met the primary endpoint of reduced incidence of G3 + ANC and improved tolerability of palbociclib administered in a 5-days-on/2-days-off weekly schedule compared to historical data from palbociclib trials^9,13,18^.

To assess target inhibition, we examined serial sTK1 activity as a surrogate pharmacodynamics marker in this trial, and demonstrated a significant reduction of sTK1 to an undetectable level in 78.2% of patients at C1D15. This result mirrors the impressive reduction in sTK1 observed 2 weeks after initiation of palbociclib in our prior study of patients with early stage HR+/HER2− breast cancer receiving neoadjuvant palbociclib and anastrozole^20^. The significant reduction of sTK1 at C1D15 and C2D1 indicates that the 5-days-on/2-days-off schedule achieved effective target inhibition and provides reassurance with the 2-day break each week.

This alternative schedule offers an advantage in avoiding the 1-week break with a 3-weeks-on/1-week-off schedule, which potentially compromises the efficacy of CDK4/6 inhibitors because of their short terminal half-life (~26 h for palbociclib^23^; 32.6 h for ribociclib^22^). In a biomarker study that assessed the longitudinal effect of palbociclib following a single dose or 3-week administration on Ki67 and pRb expression in skin biopsies and sTK1 in blood samples collected from 26 patients with HR+/HER2− MBC, recovery of pRb, Ki67, and sTK1 was observed during the off-treatment week, back to baseline on day 2–3 for pRb, day 3–4 for Ki67 and sTK1^19^. The rebound of sTK1 at the end of C1 therapy with the 3-weeks-on/1-week-off schedule was also demonstrated in the recent report by Malorni et al.^24^, in patients who received palbociclib plus fulvestrant. In contrast, the alternative 5-days-on/2-days-off schedule led to consistently profound reductions in sTK1 on C1D15 and C2D1, following the 2-day break from the previous week. In this trial, we observed a respectable CBR of 80.4% (95% CI: 66.5%–89.7%) and mPFS (95% CI) of 19.75 (12.11–34.89) months in a patient population treated in the 1st or 2nd line metastatic setting. The mPFS of 33.5 (17.25~not reached [NR]) in the endocrine sensitive population was numerically comparable to the mPFS of 27.6 months observed in the PALOMA-2^5^, with the caveat of cross-trial comparison. Similarly, the mPFS of 11.96 (10.43~NR) months observed in the endocrine-resistant population was comparable to that reported in PALOMA-3 (mPFS 11 months in the updated analysis)^25^.

In addition, our study demonstrated the potential utility of sTK1 activity at baseline and on-treatment as a prognostic marker and in monitoring disease status for patients with MBC receiving a CDK4/6 inhibitor. High sTK1 at baseline or early on-treatment time point (C1D15 or C2D1), especially at C1D15, had high degrees of accuracy in predicting progression within 6 months (84% accuracy for C1D15). In addition, we demonstrated that a rise in sTK1 predicted subsequent clinical/RECIST progression.

Our data are consistent with previous studies demonstrating that a higher baseline sTK1 is associated with a shorter time to progression in patients with advanced HR+/HER2− breast cancer receiving endocrine therapy and a rise in sTK1 on-therapy from baseline was associated with treatment resistance^26–29^. Few studies have evaluated sTK1 activity in patients receiving standard dosing palbociclib in combination with endocrine therapy. McCartney et al analyzed serial plasma TK1 activity at baseline (T0), after 1 cycle (T1), and at progression (T2) in 46 patients with HR + MBC treated with palbociclib within the TREnd trial^30^. The median TK1 activity was 75 Du/L at T0, decreased to 35 Du/L at T1, and increased to 251 Du/L at progression^30^. Patients with increasing TK1 at T1 correlated with a worse outcome than those with decreased/stable TK1 (*n* = 33; mPFS 3.0 vs 9.0 months; *p* = 0002), similar to our study^30^. Although TK1 above the median at T2 was associated with worse outcomes on post-study therapy, baseline TK1 was not prognostic^30^. In addition, in vitro studies demonstrated that TK1 reduction occurred in palbociclib sensitive but not resistant cells^30^. Cabel et al.^31^ reported a study that assessed the plasma TK1 activity at baseline and a 4-week time point in 103 patients with ER+/HER2− MBC treated with palbociclib and endocrine therapy, which demonstrated that baseline TK1 activity (using median value as cutoff) was an independent prognostic factor for both PFS and OS, but adding TK1 activity at 4 weeks did not further increase survival prediction. More recently, Malorni et al. assessed TK1 at pre-treatment, C1D15, and D28 in patients with endocrine-resistant luminal MBC receiving palbociclib and fulvestrant. In this study, TK1 was significantly suppressed on C1D15, with 90/108 (83%) patients to <20 Du/L, similar to our observation with the 5-days-on/2-days-off schedule. However, on D28, a TK1 rebound was observed in most patients in the Malorni study, with TK1 < 20 Du/L in only 29% patients, while 28 of 44 (63.6%) had TK1 < 20 Du/L on C2D1 in our study. Similar to our study, at each time point, higher TK1 was significantly and consistently associated with shorter PFS, with C1D15 being most prognostic. However, none of the prior studies assessed longitudinal TK1 activity assessment until progression. Our study is the first to show that increases in TK1 occur earlier than clinical progression, indicating detection of subclinical progression.

Our study is limited by the small sample size and the non-randomized single-arm trial design. The data regarding sTK1, at BL and early on-treatment time points, in predicting response on CDK4/6 inhibitors is intriguing as there are no predictive biomarkers currently available for CDK4/6 inhibitors. Future prospective trials are warranted to confirm our results on the safety and efficacy of the 5-days-on/2-days-off weekly schedule and to validate the clinical utility of sTK1 in guiding the management of patients with HR + /HER2- MBC.

In conclusion, this single-arm phase II trial of palbociclib administered at an alternative 5-days-on/2-days-off weekly schedule, in combination with either letrozole or fulvestrant, met the predefined primary endpoint in reducing the incidence of high-grade neutropenia, without compromising efficacy. While randomized trials are needed to confirm this finding, this alternative schedule provides an option for patients having difficulty tolerating the standard 3-weeks-on/1-week-off schedule and to potentially avoid drug discontinuation due to neutropenia. Our data demonstrate sTK1 activity a promising biomarker of prognosis and disease monitoring in patients receiving CDK4/6 inhibitors.

## Methods

### Study design and participants

A single-arm phase II study was conducted at Washington University School of Medicine, St. Louis, MO, and University of Nebraska Medical Center, Omaha, NE. Eligible patients were ≥18 years old, Eastern Cooperative Oncology Group Performance Status (ECOG PS) ≤ 2 with HR + /HER2− on the most recent biopsy, MBC with measurable or evaluable disease by Response Evaluation Criteria in Solid Tumors (RECIST) v1.1. HR+ was defined as ER+ and/or progesterone receptor positive (PR+) ≥ 1% of tumor cells. HER2- was defined per the 2013 ASCO/CAP guideline (2018 updated guideline was used for patients enrolled after May 30, 2018). Zero or one prior systemic therapies (chemotherapy or endocrine therapy) for MBC was allowed. Prior CDK4/6 inhibitor was not allowed. Laboratory requirements include absolute neutrophil count (ANC) ≥ 1500/mcl, platelets ≥100,000/mcl, total bilirubin ≤ the institutional upper limit of normal (IULN) or total bilirubin ≤3.0× IULN with direct bilirubin within the normal range in patients with Gilbert’s syndrome, AST (SGOT)/ALT (SGPT) ≤ 1.5× IULN (up to 5× IULN in patients with liver disease, Creatinine ≤ IULN or creatinine clearance ≥60 mL/min/1.73 m^2^ as determined by Cockcroft-Gault Equation for patients with creatinine > IULN. A washout of ≥3 weeks from prior chemotherapy or targeted therapy that induces myelosuppression and recovery of treatment-related AEs (except alopecia) to ≤G1 was required. All patients provided written informed consent. The study was approved by institutional review boards at the Washington University in St. Louis School of Medicine and the University of Nebraska Medical Center. The Clinicaltrials.gov# is NCT03007979.

### Treatment and procedures

Patients received palbociclib 125 mg daily, on a 5-days-on/2-days-off weekly schedule, and letrozole or fulvestrant per physician’s choice, on a 28-day cycle (C). Goserelin was administered in premenopausal patients. Complete blood count and chemistry panel were done at baseline, C1 and C2 Day (D) 15, and D1 of each cycle on C2+. Dose modifications were assessed according to guidelines in the FDA packaging insert for palbociclib, except for the use of the 5-days-on/2-days-off schedule. AEs were accessed by NCI Common Terminology Criteria for Adverse Events (CTCAE) 4.0. Patients underwent tumor evaluation by RECIST v1.1 every 3 cycles. Research blood was collected at baseline, C1D15, C2D1, and C4D1, then on D1 of every 3 cycles (with tumor imaging) until disease progression. sTK1 was analyzed retrospectively using the DiviTum^®^ assay (Biovica International, Uppsala, Sweden)^20,32^ for all patients with available samples at baseline, C1D15, C2D1, and C4D1, as well as every 3 cycles up to progression in those who progressed by the time of data cutoff. TK1 activity was determined using a refined ELISA-based method (DiviTum^®^) according to the manufacturer’s instruction (www.biovica.com) and was performed at the Biovica laboratory in Uppsala, Sweden, with laboratory investigators blinded to patient data.

### Outcomes

The primary endpoint was the rate of G3 + ANC between C1D1 and C2D1 (C1D1-29). The sample size of 47 provided 90% power, based on a one-sample binomial exact test at alpha = 5%, to test the one-sided null hypothesis of G3 + ANC rate >62%, an estimate based on incidences from prior phase III trials of palbociclib^10,13^ and that neutropenia occur early in the course of therapy^33^, versus the alternative of <40%. If G3 + ANC was observed in ≤23 patients, the 5-days-on/2-days-off schedule will be deemed as having less neutropenia than the standard schedule. As a subsequent pooled analysis of safety data from three randomized trials (PALOMA-1, 2 and 3) indicates, the rate of G3 + neutropenia in C1 in the palbociclib arm was 44.7%^18^, lower than what we originally expected. A post hoc power calculation was performed on testing against the null hypothesis H0: G3 + neutropenia rate >44.7% versus the observed 21.3% (10 out of 47, including the occurrences on C2D1 beyond C1 D1 to D28) in this trial. With *N* = 47, the post hoc power is 94.04% based on a one-sided Binomial exact test at 5% alpha level. Secondary endpoints include the rate of G3 + ANC in all cycles, palbociclib dose intensity/reduction/interruption/discontinuation, AEs, PFS for the overall population and for the endocrine sensitive or resistant population as defined by ESMO guideline^21^, objective response rate (ORR: CR + PR (complete and partial responses)) and clinical benefit rate (CBR: CR + PR + Stable disease (SD) ≥ 24 weeks by RECIST 1.1). Other endpoints include sTK1 at baseline, C1D15, C2D1, and progression, in relation to PFS and CBR, as well as, the lead time from sTK1 rise during therapy to disease progression defined by RECIST.

### Statistical analysis

Patient characteristics and AEs were summarized by descriptive statistics. AE rate, ORR, and CBR were estimated accompanied with 95% confidence interval (CI). PFS was defined from the date on treatment to off-study date (due to radiographic progression, clinical deterioration, investigator decision, AE) or date of death and to the date of the last imaging scan demonstrating no progression if patients had no events. Survival endpoints were analyzed by Kaplan–Meier (KM) method and survival difference was compared between patient groups of interests by log-rank test. Cox proportional hazard model was applied to estimate the hazard ratio (HR) with 95% CI. sTK1 ≤ 20 Du/L was deemed undetectable and was replaced with 19 Du/L for analysis. sTK1 was compared between time points by Wilcoxon signed-rank test with p values corrected by the Benjamini-Hochberg method to control false discovery rate (FDR). Baseline and on-treatment sTK1 was compared between patients who had the clinical benefit (CB) or progressive disease (PD) versus not by the Wilcoxon rank-sum test. sTK1 was dichotomized to high versus low by a pre-defined cutoff of 200 Du/L at baseline^28,29^ and 20 Du/L at on-treatment time points^24^ and was then analyzed in relation to PFS by KM method and log-rank test. Diagnostic test operating characteristics including specificity, sensitivity, negative and PPV of the dichotomized sTK1 at different time points were derived for the binary outcomes (CB vs non-CB; PD vs non-PD).

### Reporting summary

Further information on research design is available in the Nature Research Reporting Summary linked to this article.

## Supplementary information

Supplementary Material

Reporting Summary Checklist

## Data Availability

The datasets used and analyzed during the current study are available from the corresponding authors on reasonable request.

## Code availability

No code was used, as all data is provided as an individual patient or summarized data.
