# Supplementary material for: A phase II trial of an alternative schedule of palbociclib and embedded serum TK1 analysis
Source: NPJ Breast Cancer. 2022 Mar 21;8:35. doi: 10.1038/s41523-022-00399-w (PMC8938484; doi:10.1038/s41523-022-00399-w)
Supplement: Supplementary file 1 — Supplementary Material [file 41523_2022_399_MOESM1_ESM.pdf]

Supplementary Table 1. Response Summary

Best Response	N=51
CR	2
PR	14
SD ≥ 24 weeks	25
SD < 24 weeks	4
PD	6
CBR, 80.4% (95% CI 66.5-89.7%)	

Supplementary Table 2. Summary Statistics of Serum TK1 Activity (Du/L) by Time Point

Time	n	min	2.50%	25%	mean	median	sd	75%	97.50%	max
BL	51	<20	<20	42.395	389.951	97.872	704.84	490.257	1516.777	4314.002
C1D15	46	<20	<20	<20	48.845	<20	137.397	<20	168.49	936.742
C2D1	45	<20	<20	<20	145.533	<20	561.717	54.114	709.86	3725.976
C4D1	33	<20	<20	<20	122.405	<20	488.358	30.504	960.347	2803
C7D1	11	<20	<20	<20	30.312	<20	20.692	29.875	76.988	86.758
C10D1	8	<20	<20	<20	65.464	33.995	59.085	107.354	159.098	163.57
C13D1	5	<20	<20	<20	33.782	27.741	17.802	42.632	58.747	60.537
C16D1	2	<20	19.286	21.862	24.724	24.724	8.095	27.586	30.162	30.448
C19D1	2	<20	19.658	25.584	32.169	32.169	18.624	38.754	44.68	45.338
C22D1	2	<20	22.304	52.038	85.076	85.076	93.446	118.114	147.848	151.152
C25D1	1	20.711	20.711	20.711	20.711	20.711	NA	20.711	20.711	20.711
C28D1	1	103.547	103.547	103.547	103.547	103.547	NA	103.547	103.547	103.547
C32D1	1	135.558	135.558	135.558	135.558	135.558	NA	135.558	135.558	135.558
C34D1	2	<20	<20	<20	<20	<20	0	<20	<20	<20
C35D1	1	<20	<20	<20	<20	<20	NA	<20	<20	<20
C15D1	3	<20	<20	<20	<20	<20	0	<20	<20	<20
C17D1	2	<20	20.619	35.186	51.373	51.373	45.782	67.559	82.127	83.746
C3D1	3	<20	<20	<20	24.601	<20	9.701	27.401	34.962	35.802
C5D1	3	<20	20.324	32.241	626.54	45.482	1029.441	930.31	1726.655	1815.138
C6D1	2	<20	<20	<20	<20	<20	0	<20	<20	<20
C8D1	1	69.311	69.311	69.311	69.311	69.311	NA	69.311	69.311	69.311
C18D1	2	<20	<20	<20	<20	<20	0	<20	<20	<20
Progression	24	<20	<20	70.714	588.427	162.234	710.921	793.059	2234.666	2342.238

Supplementary Table 3. Comparison of Serum TK1 Activity between Two Time Points.

Comparison	time1	time2	% samples with Decreased TK1 (time 2 vs. time1)	Wilcoxon signed rank test P	FDR P
C1D15 vs. BL	BL	C1D15	84.78261	5.47E-08	4.03E-07
C2D1 vs. BL	BL	C2D1	86.36364	8.06E-08	4.03E-07
C4D1 vs. BL	BL	C4D1	81.81818	0.0001	0.000251
Progression vs. BL	BL	Progression	50	0.330505	0.367228
C2D1 vs. C1D15	C1D15	C2D1	12.82051	0.263494	0.329368
C4D1 vs. C1D15	C1D15	C4D1	3.571429	0.010827	0.018045
Progression vs. C1D15	C1D15	Progression	0	6.41E-05	0.000214
C4D1 vs. C2D1	C2D1	C4D1	22.22222	0.888841	0.888841
Progression vs. C2D1	C2D1	Progression	4.347826	0.000301	0.000601
Progression vs. C4D1	C4D1	Progression	7.692308	0.021484	0.030692

sTK1 activity was significantly decreased at the 3 early time points (C1D15, C2D1, cycle 4) compared to BL. sTK1 activity was significantly increased at progression compared to C1D15, C2D1, C4D1. sTK1 activity at C4D1 was significantly increased compared to C1D15.

Supplementary Table 4. Time point and Days to Progression from Baseline by sTK1 or by Clinical/RECIST criteria (N=24)

PID	Time point of TK1 progression	Time to TK1 progression (Days)	Time to Clinical Progression (Days)	Lead Time
PAD002	C10D1	274	974	-700
PAD003	C2D1	28	91	-63
PAD005	Progression	88	88	0
PAD007	C4D1	91	343	-252
PAD009	Progression	81	81	0
PAD011	C7D1	171	329	-158
PAD012	No increase	NA	87	NA
PAD014	Progression	973	973	0
PAD017	Progression	337	337	0
PAD021	C2D1	35	516	-481
PAD022	C4D1	84	686	-602
PAD025	Progression	85	85	0
PAD026	C4D1	84	168	-84
PAD027	C2D1	35	90	-55
PAD029	C8D1	181	252	-71
PAD033	Progression	41	41	0
PAD034	C2D1	28	217	-189
PAD035	Progression	581	581	0
PAD040	C2D1	28	84	-56
PAD041	Progression	588	588	0
PAD042	No increase	NA	81	NA
PAD043	C13D1	361	573	-212
PAD045	Progression	342	342	0
PADUNMC002	C2D1	35	498	-463

The 24 patients who had disease progressed are listed. TK1 progression is defined as the earliest time point with an increase in sTK1 compared to a previous consecutive time point.

Supplementary Table 5. Summary Statistics of sTK1 (Du/L) at BL or C1D15 in Subgroups

Group	n	min	2.50%	25%	mean	median	sd	75%	97.50%	max
Baseline sTK1 (Du/L) Summary Statistics in Subgroups										
CR	2	29.889	31.589	46.885	63.88	63.88	48.071	80.876	96.172	97.872
PR	14	<20	<20	24.743	145.737	52.838	227.378	107.711	696.415	755.858
SD	29	<20	<20	44.471	481.53	140.952	865.937	523.015	2361.026	4314.002
PD	6	71.981	83.782	174.293	625.846	550.505	555.909	972.543	1366.941	1419.972
CB=NO	10	71.981	93.223	211.394	1116.266	949.362	1254.701	1394.479	3686.26	4314.002
CB=YES	41	<20	<20	29.889	212.802	61.288	320.528	185.788	952.146	1495
Endocrine sensitive	29	<20	<20	45.673	309.757	97.872	433.884	457.499	1451.191	1524.036
Endocrine resistant	22	<20	<20	34.903	495.663	125.466	954.6	503.594	2834.026	4314.002
Visceral=NO	25	<20	<20	40.32	407.433	61.288	874.666	523.015	2323.035	4314.002
Visceral=YES	26	<20	<20	54.262	373.142	105.295	508.489	454.457	1505.889	1524.036
C1D15 sTK1 (Du/L) Summary Statistics in Subgroups										
CR	2	<20	<20	<20	<20	<20	<20	<20	<20	<20
PR	13	<20	<20	<20	19.311	<20	1.12	<20	21.826	23.037
SD	25	<20	<20	<20	35.079	<20	40.913	<20	161.204	170.407
PD	6	<20	<20	<20	180.144	24.5	370.95	50.342	826.79	936.742
CB=NO	9	<20	<20	<20	164.435	57.123	295.356	155.069	783.475	936.742
CB=YES	37	<20	<20	<20	20.729	<20	7.682	<20	32.327	64.891
Endocrine sensitive	27	<20	<20	<20	61.015	<20	177.566	<20	438.624	936.742
Endocrine resistant	19	<20	<20	<20	31.552	<20	33.373	<20	118.398	155.069
Visceral=NO	22	<20	<20	<20	22.819	<20	12.418	<20	60.813	64.891
Visceral=YES	24	<20	<20	<20	72.703	<20	188.553	24.667	496.099	936.742

CR, complete response; PR, partial response; SD, stable disease; PD, progression by RECIST; CB, clinical benefit; Visceral, mets in visceral organs.

Supplementary Table 6. Correlation of levels of sTK1 (Du/L) at BL or C1D15 with Clinical Benefit on therapy, Visceral Mets, or Endocrine Sensitivity

Variable	sTK1 Time point	Wilcoxon Rank Sum test P
Clinical Benefit (Yes vs No)	BL	0.000419
Clinical Benefit (Yes vs No)	C1D15	8.40E-05
Visceral mets (Yes vs No)	BL	0.527376
Visceral mets (Yes vs No)	C1D15	0.053003
Endocrine Sensitive vs Resistant	BL	0.76787
Endocrine Sensitive vs Resistant	C1D15	0.963016

Supplementary Table 7a. Sensitivity, Specificity, PPV and NPV of High sTK1 at BL or early time points for not achieving clinical benefit (progression within 6 cycles) or PD as the best response (progression within 3 cycles).

High sTK1 in predicting not achieving clinical benefit (progression within 6 cycles)								
Timepoint	Prevalence	Specificity	Sensitivity	FN	FP	NPV	PPV	Accuracy
BL	0.196078	0.780488	0.7	0.3	0.219512	0.914286	0.4375	0.764706
C1D15	0.195652	0.891892	0.666667	0.333333	0.108108	0.916667	0.6	0.847826
C2D1	0.227273	0.735294	0.7	0.3	0.264706	0.892857	0.4375	0.727273
High sTK1 in predicting PD as the best response (progression within 3 cycles)								
Timepoint	Prevalence	Specificity	Sensitivity	FN	FP	NPV	PPV	Accuracy
BL	0.117647	0.711111	0.5	0.5	0.288889	0.914286	0.1875	0.686275
C1D15	0.130435	0.825	0.5	0.5	0.175	0.916667	0.3	0.782609
C2D1	0.136364	0.657895	0.5	0.5	0.342105	0.892857	0.1875	0.636364

Cutoff for high sTK1 is 200 Du/L at baseline (BL) and 20 Du/L at on-treatment time points (C1D15, C2D1, C4D1). FN, false negative; FP, false positive; NPV, negative predictive value; PPV, positive predictive value.

Supplementary Table 7b. 2x2 Table for Suppl. Table S7A

Variable	sTK1 Levels	All N (%)	CB=N N (%)	CB=Y N (%)	PD=N N (%)	PD=Y N (%)
BL (N=51)						
	High sTK1	16 (31.37)	7 (70)	9 (21.95)	13(28.89)	3(50)
	Low sTK1	35 (68.63)	3 (30)	32 (78.05)	32(71.11)	3(50)
C1D15 (N=46)						
	High sTK1	10 (21.74)	6 (66.67)	4 (10.81)	7(17.5)	3(50)
	Low sTK1	36 (78.26)	3 (33.33)	33 (89.19)	33(82.5)	3(50)
C2D1 (N=44)						
	High sTK1	16 (36.36)	7 (70)	9 (26.47)	13(34.21)	3(50)
	Low sTK1	28 (63.64)	3 (30)	25 (73.53)	25(65.79)	3(50)

Supplementary Table 8. Multivariate analysis

Variable	BL sTK1 HR (95% CI)	p	C1D15 sTK1 HR (95% CI)	p	C2D1 sTK1 HR (95% CI)	p
sTK1 (high vs.low)*	3.02 (1.35~6.73)	**0.0069**	3.3 (1.24~8.76)	**0.0168**	2.9 (1.28~6.57)	**0.01058**
Age (continuous)	1.01 (0.97~1.05)	0.6608	1 (0.97~1.04)	0.8926	1 (0.96~1.04)	0.948687
endo Sensitive (No vs.Yes)	2.57 (1.05~6.29)	**0.0382**	2.45 (1~5.98)	**0.0490**	2.84 (1.06~7.63)	**0.03875**
Visceral mets (Yes vs. No)	2.26 (1.05~4.89)	**0.0378**	1.58 (0.65~3.81)	0.3087	2.63 (1.12~6.19)	**0.026452**
Bone mets (Yes vs. No)	1.35 (0.44~4.16)	0.5963	1.42 (0.47~4.31)	0.5316	1.85 (0.62~5.54)	0.269091

* high/low by cutoff of 200 at BL and by cutoff of 20 at other time points

Suppl. Fig 1 Consort Diagram

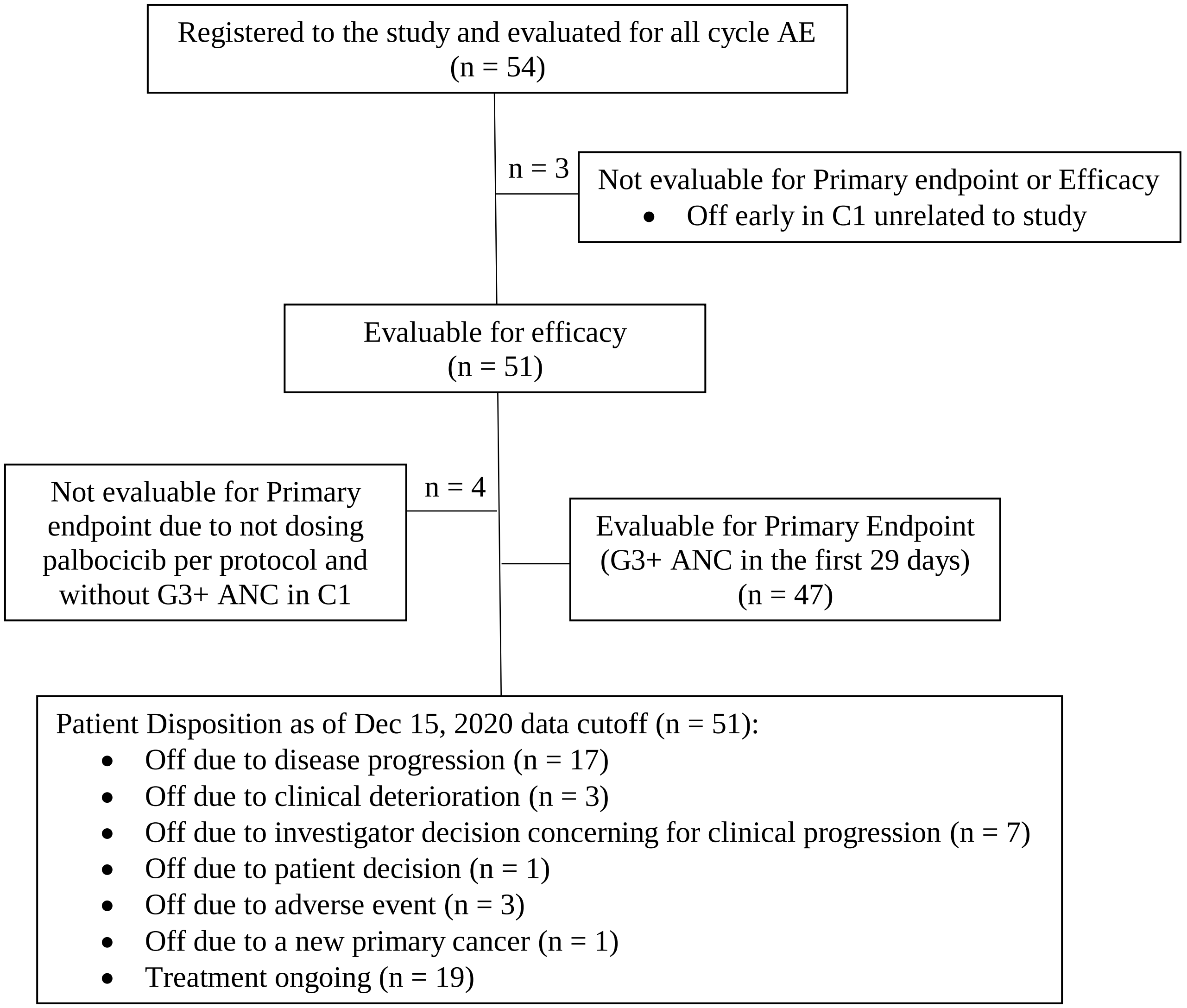

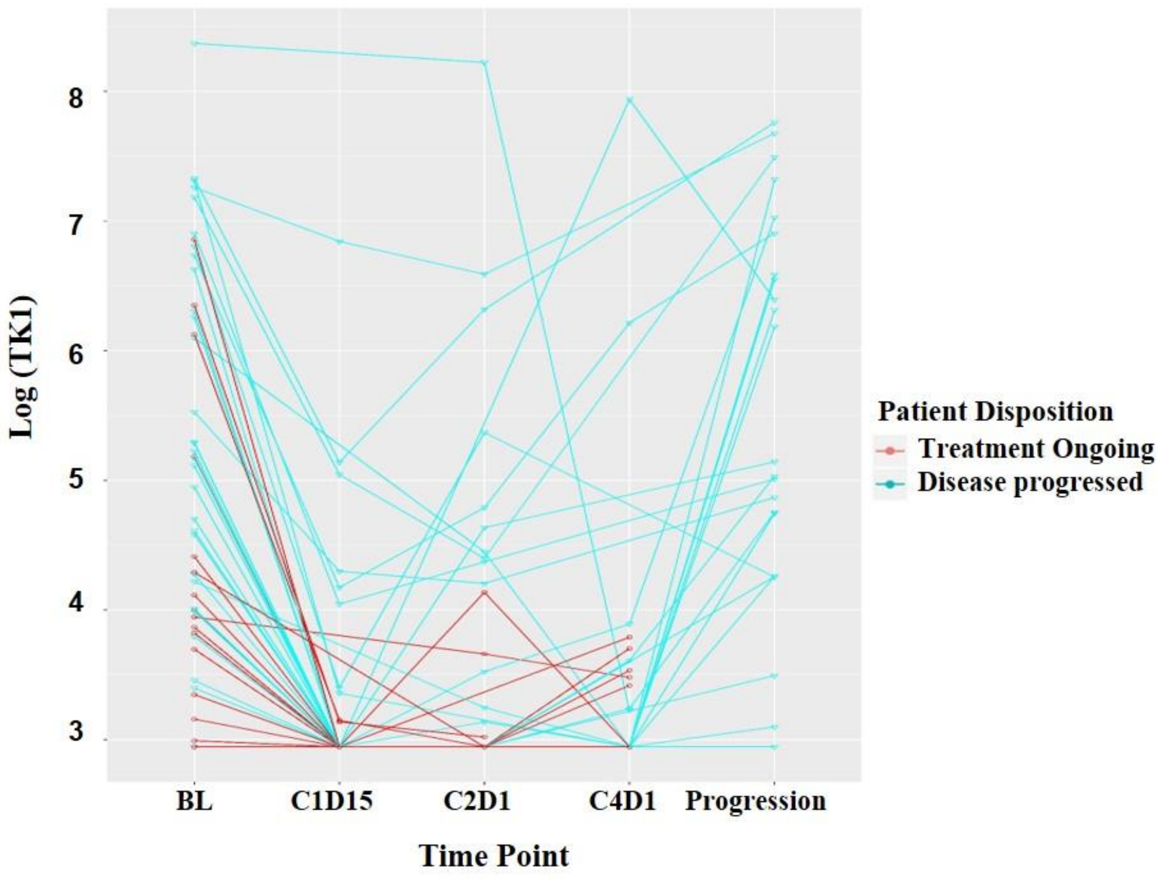

Supplementary Figure 2. Longitudinal plot of log(TK1) at different time points for individual patients. Line color indicates whether progression has occurred or not. BL, baseline; C1D15, cycle 1 day 15; C2D1, cycle 2 day 1; Progression, PFS events that included RECIST progression (n=17) and investigator decision (n=7).

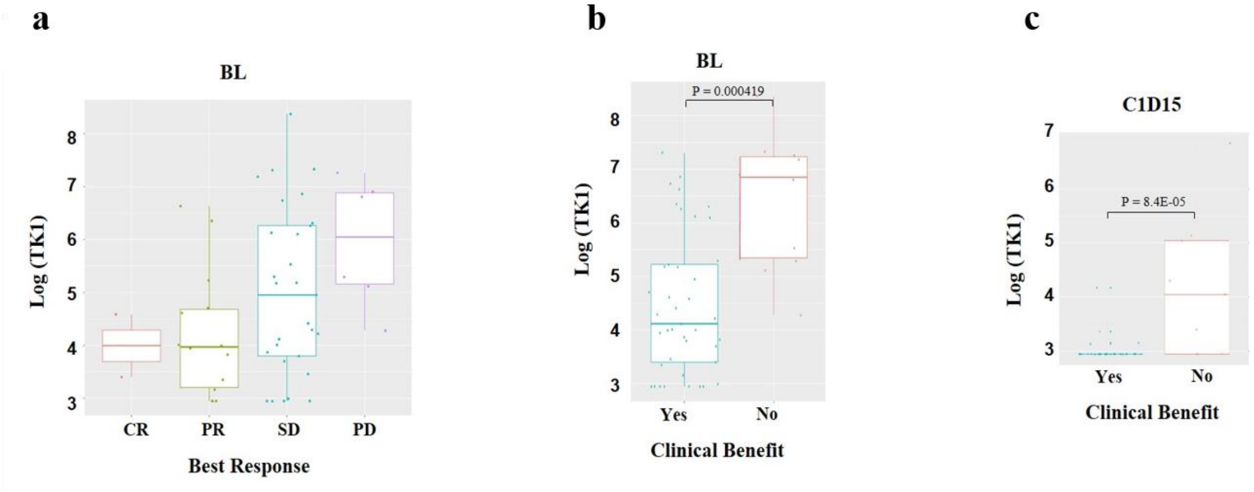

Supplementary Figure 3. Serum TK1 activity in relation to disease status on treatment. (a) Box plot of baseline serum log(TK1) by RECIST best response category; (b) Box plot of baseline serum log(TK1) by achieving clinical benefit or not; (c) Box plot of C1D15 serum log(TK1) by achieving clinical benefit or not. CR, complete response; PR, partial response; SD, stable disease; PD, progression by RECIST; Clinical benefit, defined as CR, or PR, or SD ≥24 weeks. The center horizontal line of the box-plot indicates the median, while the box limits indicate the upper and lower quartiles. The box-plot whiskers show a 1.5x interquartile range. Those points outside the whisker line are indicated as outliers.
