# Supplementary material for: A phase II trial of an alternative schedule of palbociclib and embedded serum TK1 analysis
Source: NPJ Breast Cancer. 2022 Mar 21;8:35. doi: 10.1038/s41523-022-00399-w (PMC8938484; doi:10.1038/s41523-022-00399-w)
Supplement: Supplementary file 2 — Reporting Summary Checklist [file 41523_2022_399_MOESM2_ESM.pdf]

nature portfolio

Corresponding author(s): Jairam Krishnamurthy
Cynthia X. Ma

Last updated by author(s): Nov 4, 2021

Reporting Summary

Nature Portfolio wishes to improve the reproducibility of the work that we publish. This form provides structure for consistency and transparency in reporting. For further information on Nature Portfolio policies, see our Editorial Policies and the Editorial Policy Checklist.

Statistics

For all statistical analyses, confirm that the following items are present in the figure legend, table legend, main text, or Methods section.

n/a	Confirmed	
☐	☒	The exact sample size (n) for each experimental group/condition, given as a discrete number and unit of measurement
☒	☐	A statement on whether measurements were taken from distinct samples or whether the same sample was measured repeatedly
☐	☒	The statistical test(s) used AND whether they are one- or two-sided<br>*Only common tests should be described solely by name; describe more complex techniques in the Methods section.*
☐	☒	A description of all covariates tested
☐	☒	A description of any assumptions or corrections, such as tests of normality and adjustment for multiple comparisons
☐	☒	A full description of the statistical parameters including central tendency (e.g. means) or other basic estimates (e.g. regression coefficient) AND variation (e.g. standard deviation) or associated estimates of uncertainty (e.g. confidence intervals)
☐	☒	For null hypothesis testing, the test statistic (e.g. F, t, r) with confidence intervals, effect sizes, degrees of freedom and P value noted<br>*Give P values as exact values whenever suitable.*
☒	☐	For Bayesian analysis, information on the choice of priors and Markov chain Monte Carlo settings
☒	☐	For hierarchical and complex designs, identification of the appropriate level for tests and full reporting of outcomes
☒	☐	Estimates of effect sizes (e.g. Cohen's d, Pearson's r), indicating how they were calculated

Our web collection on statistics for biologists contains articles on many of the points above.

Software and code

Policy information about availability of computer code

Data collection	No software was used.
Data analysis	No software was used.

For manuscripts utilizing custom algorithms or software that are central to the research but not yet described in published literature, software must be made available to editors and reviewers. We strongly encourage code deposition in a community repository (e.g. GitHub). See the Nature Portfolio guidelines for submitting code & software for further information.

Data

Policy information about availability of data

All manuscripts must include a data availability statement. This statement should provide the following information, where applicable:

- Accession codes, unique identifiers, or web links for publicly available datasets
- A description of any restrictions on data availability
- For clinical datasets or third party data, please ensure that the statement adheres to our policy

The datasets used and analyzed during the current study are available from the corresponding author on reasonable request.

Field-specific reporting

Please select the one below that is the best fit for your research. If you are not sure, read the appropriate sections before making your selection.

☒ Life sciences ☐ Behavioural & social sciences ☐ Ecological, evolutionary & environmental sciences

For a reference copy of the document with all sections, see nature.com/documents/nr-reporting-summary-flat.pdf

Life sciences study design

All studies must disclose on these points even when the disclosure is negative.

Sample size

The sample size of 47 provided 90% power, based on one-sample binomial exact test at alpha=5%, to test the 1-sided null hypothesis of G3+ ANC rate >62%, an estimate based on incidences from prior phase III trials of palbociclib10,13 and that neutropenia occur early in the course of therapy33, versus the alternative of <40%. If G3+ ANC was observed in ≤23 patients, the 5-days-on/2-days-off schedule will be deemed as having less neutropenia than the standard schedule. As a subsequent pooled analysis of safety data from 3 randomized trials (PALOMA-1, 2 and 3) indicates, the rate of G3+ neutropenia in C1 in the palbociclib arm was 44.7%18, lower than what we original expected. A post-hoc power calculation was performed on testing against the null hypothesis H0: G3+ neutropenia rate >44.7% versus the observed 21.3% (10 out of 47, including the occurrences on C2D1 beyond C1 D1 to D28) in this trial. With N=47, the post-hoc power is 94.04% based on 1-sided Binomial exact test at 5% alpha level.

Data exclusions

51 of 54 patients who were enrolled were evaluable for adverse event and clinical benefit assessments and completed at least 1 cycle of therapy.

Replication

Findings from our trial were not replicated, but were compared to historical trials with Palbociclib.

Randomization

This trial is a single-arm, not randomized Phase II study. All patients received Palbociclib with the addition of fulvestrant or letrozole per the choice of the physician.

Blinding

This trial was not randomized therefore blinding of participants and investigators was unnecessary.

Reporting for specific materials, systems and methods

We require information from authors about some types of materials, experimental systems and methods used in many studies. Here, indicate whether each material, system or method listed is relevant to your study. If you are not sure if a list item applies to your research, read the appropriate section before selecting a response.

Materials & experimental systems

n/a	Involved in the study	
☒	☐	Antibodies
☒	☐	Eukaryotic cell lines
☒	☐	Palaeontology and archaeology
☒	☐	Animals and other organisms
☐	☒	Human research participants
☐	☒	Clinical data
☒	☐	Dual use research of concern

Methods

n/a	Involved in the study	
☒	☐	ChIP-seq
☒	☐	Flow cytometry
☒	☐	MRI-based neuroimaging

Human research participants

Policy information about studies involving human research participants

Population characteristics

Trial participants were characterized as hormone receptor-positive and HER2-negative with advanced/metastatic breast cancer. The median age of participants was 61 years with 83% being White and 17% Black.

Recruitment

The Principle Investigator and the engaged list participating physicians recruited patients from their individual oncology clinics.

Ethics oversight

The IRB of the Washington University in St. Louis Human Research Protection Office reviewed, approved and had oversight of this trial.

Note that full information on the approval of the study protocol must also be provided in the manuscript.

Clinical data

Policy information about clinical studies

All manuscripts should comply with the ICMJE guidelines for publication of clinical research and a completed CONSORT checklist must be included with all submissions.

Clinical trial registration	NCT3007979
Study protocol	The trial protocol is available upon reasonable request.
Data collection	54 patients were enrolled between July 12, 2017 and Feb 14, 2020 from the clinics of participating investigators. Patient data was collected, de-indentified and stored in a secure database.
Outcomes	The primary endpoint was the rate of G3+ ANC between C1D1 and C2D1 (C1D1-29). The sample size of 47 provided 90% power, based on one-sample binomial exact test at alpha=5%, to test the 1-sided null hypothesis of G3+ ANC rate >62%, an estimate based on incidences from prior phase III trials of palbociclib10,13 and that neutropenia occur early in the course of therapy33, versus the alternative of <40%. If G3+ ANC was observed in ≤23 patients, the 5-days-on/2-days-off schedule will be deemed as having less neutropenia than the standard schedule. As a subsequent pooled analysis of safety data from 3 randomized trials (PALOMA-1, 2 and 3) indicates, the rate of G3+ neutropenia in C1 in the palbociclib arm was 44.7%18, lower than what we original expected. A post-hoc power calculation was performed on testing against the null hypothesis H0: G3+ neutropenia rate >44.7% versus the observed 21.3% (10 out of 47, including the occurrences on C2D1 beyond C1 D1 to D28) in this trial. With N=47, the post-hoc power is 94.04% based on 1-sided Binomial exact test at 5% alpha level. Secondary endpoints include the rate of G3+ ANC in all cycles, palbociclib dose intensity/reduction/interruption/discontinuation, AEs, PFS for the overall population and for endocrine sensitive or resistant population as defined by ESMO guideline21, objective response rate (ORR: CR+PR (complete and partial responses)) and clinical benefit rate (CBR: CR+PR+Stable disease (SD) ≥24 weeks by RECIST 1.1).
